# Neurovascular and neuroimaging effects of the hallucinogenic serotonin receptor agonist psilocin in the rat brain

**DOI:** 10.1016/j.neuropharm.2015.07.018

**Published:** 2015-12

**Authors:** Aisling Spain, Clare Howarth, Alexandre A. Khrapitchev, Trevor Sharp, Nicola R. Sibson, Chris Martin

**Affiliations:** a Department of Psychology, University of Sheffield, Western Bank, Sheffield S10 2TP, UK; b Cancer Research UK & Medical Research Council Oxford Institute for Radiation Oncology, Department of Oncology, University of Oxford, Oxford OX3 7DQ, UK; c Department of Pharmacology, University of Oxford, Mansfield Road, Oxford OX1 3QT, UK

**Keywords:** Psilocin, Psilocybin, Pharmacological MRI, Functional MRI, Neurovascular, Cerebral haemodynamics, AUC, area under the curve, BOLD, blood oxygen level dependent, CBF, cerebral blood flow, ISI, inter-stimulus-interval, LC, locus coeruleus, LFP, local field potential, phMRI, pharmacological MRI, ROI, region of interest, SSRI, selective serotonin reuptake inhibitor

## Abstract

The development of pharmacological magnetic resonance imaging (phMRI) has presented the opportunity for investigation of the neurophysiological effects of drugs *in vivo*. Psilocin, a hallucinogen metabolised from psilocybin, was recently reported to evoke brain region-specific, phMRI signal changes in humans. The present study investigated the effects of psilocin in a rat model using phMRI and then probed the relationship between neuronal and haemodynamic responses using a multimodal measurement preparation. Psilocin (2 mg/kg or 0.03 mg/kg i.v.) or vehicle was administered to rats (N = 6/group) during either phMRI scanning or concurrent imaging of cortical blood flow and recording of local field potentials. Compared to vehicle controls psilocin (2 mg/kg) evoked phMRI signal increases in a number of regions including olfactory and limbic areas and elements of the visual system. PhMRI signal decreases were seen in other regions including somatosensory and motor cortices. Investigation of neurovascular coupling revealed that whilst neuronal responses (local field potentials) to sensory stimuli were decreased in amplitude by psilocin administration, concurrently measured haemodynamic responses (cerebral blood flow) were enhanced. The present findings show that psilocin evoked region-specific changes in phMRI signals in the rat, confirming recent human data. However, the results also suggest that the haemodynamic signal changes underlying phMRI responses reflect changes in both neuronal activity and neurovascular coupling. This highlights the importance of understanding the neurovascular effects of pharmacological manipulations for interpreting haemodynamic neuroimaging data.

## Highlights

• The pharmacological MRI (phMRI) response to psilocin is characterised in rats.
• Region specific increases and decreases in phMRI signals are observed.
• Underlying neuronal and haemodynamic changes are also measured *in vivo*.
• Evidence for altered neuronal-haemodynamic (neurovascular) coupling by psilocin.
• phMRI interpretation should consider neurovascular as well as neuronal drug effects.

## 1 Introduction

Pharmacological MRI (phMRI) offers the opportunity for *in vivo* characterisation of the neurophysiological effects of drugs on the brain. As such, it is becoming an increasingly important tool in basic research, drug discovery and development (Borsook et al., 2006, Murphy and Mackay, 2011, Wise and Tracey, 2006), and in research studies involving clinical populations (Fleisher et al., 2009, Mukherjee et al., 2014, Reynell and Harris, 2013). As it is a technique that can be deployed in both human and experimental animal research paradigms, phMRI is also finding an important translational role in neuropsychopharmacological research (Couch et al., 2013, Schwarz et al., 2007). This approach has recently been applied to psilocybin, the active constituent of “magic” mushrooms; which is of interest due to its potential utility in treating affective disorders (Carhart-Harris et al., 2012a, Carhart-Harris et al., 2012b, Grob et al., 2011, Vollenweider and Kometer, 2010).

Psilocybin, similar to other hallucinogenic agents, is an agonist at the serotonin 2A (5-HT_2A_) receptor, which is the primary mediator of its hallucinogenic effects (Quednow et al., 2012, Vollenweider et al., 1998) but whether and how this action is relevant to its therapeutic effects is unclear. It also shows affinity for all other serotonin receptors, with the exception of 5-HT_3_ (Halberstadt and Geyer, 2011). 5-HT_2A_ receptor agonism is known to modulate pyramidal cell activity in the prefrontal cortex and anterior cingulate cortex, areas implicated in affective disorders (Vollenweider and Kometer, 2010). Positron emission tomography (PET) studies in humans show changes in cerebral glucose metabolism in the anterior cingulate cortex as well as in other frontal areas associated with cognitive changes following psilocybin administration (Gouzoulis-Mayfrank et al., 1999, Vollenweider et al., 1997) while widespread decreases in cerebral blood flow (CBF) and phMRI signals have been observed (Carhart-Harris et al., 2012a).

Findings such as those described above are potentially important for guiding the development of therapeutic drugs, as well as for refining our understanding of brain disease. However, interpretation of phMRI data is not straightforward, as the haemodynamic changes upon which the imaging signals depend, such as the BOLD response, are not a direct measure of neuronal activity, but rather rely on the relationship between haemodynamic changes and the underlying neuronal activity (Logothetis, 2008). In pharmacological neuroimaging, this relationship, known as neurovascular coupling, may be affected by both the pharmacological manipulations themselves and/or by resultant alterations in neurotransmission (Martin and Sibson, 2008). This means that phMRI studies cannot solely be interpreted in terms of the effects of the drug of interest upon neurons.

In the case of psilocybin, whilst the pronounced phMRI signal decreases observed could be interpreted as evidence of neuronal deactivation (Carhart-Harris et al., 2012a), the physiological mechanisms producing decreased phMRI signals, and their relationship to neuronal activity remains unclear. Indeed, such processes may be highly dependent on differences between anatomical regions, and local synaptic input, as well as a number of factors (Kim and Ogawa, 2012, Lauritzen et al., 2012).

*In vivo*, psilocybin undergoes first pass metabolism to psilocin, the active metabolite thought to account for most of the psychotropic effects of psilocybin administration (Hasler et al., 1997, Passie et al., 2002). A further potential confound in interpretation of BOLD signals in the case of psilocin is the combined neuronal and vascular effects of serotonergic drugs (Cohen et al., 1996, Fukuda et al., 2002). The 5-HT_2A_ receptor has vasoconstrictive effects (Kovács et al., 2012, Martin, 1994), and psilocin also has affinity for the 5-HT_1D_ and 5-HT_1B_ receptors (Halberstadt and Geyer, 2011), which have both neuronal and vascular effects (Gupta and Villalón, 2010, Kovács et al., 2012). Additionally, serotonergic innervation of the cerebrovasculature may result in vascular effects of serotonin drugs that are separate from their direct actions on neuronal receptors (Hamel, 2006).

In experimental animal models, it is possible to combine neuronal recordings with simultaneous measurement of haemodynamics in order to better characterise the source of the negative BOLD signal and investigate in fine detail the coupling between neuronal and haemodynamic signal changes (Boorman et al., 2010). Evidence suggests negative BOLD signals can have separable haemodynamic (Devor et al., 2005, Harel et al., 2002) and neuronal (Shmuel et al., 2006) sources and may occur in the presence of increased neuronal signalling (Angenstein et al., 2009). Furthermore, in cases where negative BOLD signals are associated with reduced neuronal responses it is difficult in a phMRI paradigm to distinguish between direct drug effects and the effects of activation of inhibitory interneurons (Shih et al., 2009). In summary, a more detailed understanding of haemodynamic-based neuroimaging signals in the context of pharmacological manipulations is important for the continued application of phMRI techniques in human subjects.

The aim of this study was to determine the effects of pharmacological manipulation of serotonergic neurotransmission by psilocin on the relationship between neuronal activity and the haemodynamic responses that underpin neuroimaging signal changes. We used a rodent model in which we combined whole brain phMRI measures of drug action with determination of neurovascular coupling relationships by concurrent measurement of neuronal activity and cerebral blood flow changes in response to sensory stimulation.

## 2 Materials and methods

### 2.1 Animals and experimental design

Male Sprague–Dawley rats (Charles River, UK) weighing 213–381 g were used. Animals were housed under a 12:12 h light/dark cycle, with food and water available *ad libitum*. All experiments were carried out in accordance with the UK Animals (Scientific Procedures) Act (1986) under a U.K. Home Office licence. Eighteen Animals were used for phMRI data acquisition, comprising vehicle control, low-dose and high-dose groups (N = 6 per group). A further 12 animals were used for concurrent cerebral blood flow and neuronal activity measures, comprising drug and vehicle control groups (N = 6 per group) in a repeated measures design (pre and post-drug stimulation and data acquisition epochs).

### 2.2 Surgical procedures

Animals were anaesthetised with 4–5% isoflurane in a 30:70 mixture of oxygen and nitrogen, and maintained on 2–3% isoflurane for the duration of surgical procedures. Animals were tracheotomised and the femoral vein and artery cannulated. To record cerebral blood flow, high resolution laser speckle contrast imaging was conducted. The animal's head was fixed in a stereotaxic frame and a window in the cranium was thinned over the left somatosensory cortex. To record neuronal activity, a burr hole was drilled in the rostrolateral corner of the thinned cranial window and a recording electrode was inserted to a depth of 0.5 mm. Following surgery, animals were maintained on 1.5% isoflurane anaesthesia for the remainder of the experiment. At the end of all experiments, animals were killed by overdose with pentobarbital or transcardially perfused under terminal anaesthesia.

### 2.3 Drugs

Psilocin (Lipomed, Arlesheim, Switzerland and Tocris, Abingdon, UK) was dissolved in 0.05 mM tartaric acid vehicle and 1N sodium hydroxide used to raise the pH to 5–7. Final concentrations were 1 mg/mL, for injections at a dose of 2 mg/kg, and 0.075 mg/mL for injections at a dose of 0.03 mg/kg.

### 2.4 Functional MRI experiments

Functional MRI acquisitions were carried out on a 9.4 T horizontal bore MRI system (Agilent Technologies, UK) using a 72 mm volume coil (InsightMRI, Worcester MA, USA; m2m Imaging, Cleveland OH, USA and RAPID Biomedical, Rimpar, Germany). Datasets of 26 horizontal slices to cover the whole brain were acquired using a T2*-weighted multi-echo gradient-echo sequence with the following parameters: flip angle = 15°; matrix size = 96 × 96; relaxation time (TR) = 625 ms; echo time (TE) = 3–18 ms (6 echos, 3 ms apart); slice thickness = 0.5 mm. One volume was acquired each minute. Baseline datasets (15 min) were acquired before i.v. administration of psilocin (2 mg/kg or 0.03 mg/kg) or vehicle (N = 6/group), and imaging continued for 45 min after drug administration. Arterial blood gases were sampled before and after imaging.

### 2.5 Cerebral blood flow and neuronal activity measurements

The laser speckle contrast imager (FLPI-2, Moor Instruments, Axminster, UK; 785 nm laser diode; 576 × 768 pixel greyscale CCD image capture) was positioned above the thinned cranial window and blood flow was imaged at 25 Hz and recorded using moorFLPI Measurement v3.0 (Moor Instruments, UK). Local field potentials (LFPs) were simultaneously acquired from the implanted electrode (0.155 mm diameter, Teflon insulated platinum; Bilaney Consultants Ltd., Sevenoaks, UK), sampled at 10 kHz using an isolated amplifier unit coupled to a data acquisition device (systems 1902 and 1401, Cambridge Electronic Design, Cambridge, UK), and recorded using a PC running Spike2 software (Cambridge Electronic Design, UK), which also controlled stimulus delivery. There was a minimum 45 min delay before the start of recordings to avoid the influence of cortical spreading depression effects following electrode implantation.

### 2.6 Stimulation protocol

Prior to administration of 2 mg/kg psilocin or vehicle (N = 6/group) animals underwent 10 whisker pad stimulation trains of 16 s duration, with a 60 s inter-stimulus-interval (ISI), at a frequency of 10 Hz. Following this, 60 stimulation trains of 2 s duration, with a 25 s ISI, at frequencies of 1, 2, 5, 10, 20 and 40 Hz were carried out. The order of stimulus frequencies was pseudorandomised. In all cases the amplitude of the stimulus was 1.2 mA and the stimulation pulse width was 0.3 ms. Subsequently, four 30 s duration episodes of hypercapnia (10% CO_2_) were induced, with an interval of 3 min between the start of each episode. After drug administration the stimulation and hypercapnia paradigms were repeated as before.

### 2.7 Data analysis

fMRI data were averaged across echoes prior to analysis in order to increase the contrast-to-noise ratio. Data were analysed using FEAT (FMRI Expert Analysis Tool) Version 5.92, part of FMRIB's Software Library (FSL, www.fmrib.ox.ac.uk/fsl; Jenkinson et al., 2012). For analysis of individual animals, data were smoothed, the time of drug administration used as a regressor in the general linear model with the scan registered to a standard template (Schweinhardt et al., 2003). Activated voxels were identified using a cluster thresholding method (Worsley et al., 1992) with a z-score threshold of 2.3 and cluster significance level of 0.05. Individual animal analyses were combined for higher level analysis using a fixed effects model to compare the effects of the two doses of psilocin to placebo, and to generate mean change in BOLD activation for each group. Regions of interest (ROIs) for time series extraction were defined as areas showing significant BOLD signal change in animals that received 2 mg/kg psilocin within the following anatomical ROIs based on the changes in human subjects seen in Carhart-Harris et al., (2012a); the hypothalamus, amygdala and cingulate cortex. The somatosensory cortex was also included. Extracted time series were normalised to the baseline 15 min of pre-drug administration scanning and averaged in 5 min bins.

Processing of laser speckle and LFP data was carried out using custom-written code in Matlab (2013a), and SPSS 21 was used for statistical analysis. Laser speckle data were downsampled to 5 Hz. For CBF value changes in response to whisker stimulation, statistical parametric mapping was used to identify a responsive ROI. Time series data from this region were extracted and normalised to a 10 s baseline period preceding each stimulation train to give percentage changes in cerebral blood flow. The area under the curve (AUC) and maximum for each response were calculated. Where stimulation trains were of 16 s duration, both early (0–8 s after stimulation onset) and late (10–20 s after stimulation onset) response maxima were identified. The response maxima for each animal (pre- and post-drug treatment) were divided by the pre-drug maxima to give a measure of fractional change in response magnitude. Statistical comparisons were made using multifactorial ANOVA where pre- and post-drug administration, drug type and stimulation frequency (where applicable) were used as factors.

For analysis of data from hypercapnic challenge, the entire thinned skull area was used as an ROI and the extracted CBF data were normalised to a 25 s baseline prior to the onset of each CO_2_ challenge. Data were normalised as for whisker stimulation experiments and smoothed using a Savitsky–Golay filter. Statistical comparisons were by two-way ANOVA, where pre- and post-drug administration and drug type were used as factors and a *p* value of less than 0.05 was considered significant.

In electrophysiological recordings the stimulation artefact was removed from the LFP response to each pulse in each stimulation episode. The stimulation artefact was replaced by a straight line vector containing the same number of points as the stimulation artefact and connecting the last point before and the first point after the artefact. The baseline of the response to each stimulation train was set to zero by subtracting the mean calculated over 100 ms prior to stimulation onset. Data were band-pass filtered (pass between 17.36 Hz and 2604.25 Hz) to remove low and high frequency noise. To quantify neuronal response magnitudes, the AUC was calculated for each pulse in the stimulation train (over a 20.5 ms period following stimulation onset) and values were summed over the stimulation train. Statistical comparisons were made as for the CBF responses to stimulation.

One animal in the vehicle-treated group was excluded from these analyses as it showed a maximal CBF response to stimulation before drug administration that was more than eleven standard deviations greater than the mean maximum response seen in other animals in the group.

## 3 Results

### 3.1 Functional MRI

Psilocin, at a dose of 2 mg/kg, produced a mixed pattern of positive and negative BOLD signal changes throughout the brain compared to vehicle treated controls. A summary of the regions showing signal changes is presented in Table 1. At a dose of 0.03 mg/kg psilocin produced a small BOLD signal decrease in the cerebellum compared to vehicle treated controls but had no effect in other regions. Consequently, experiments carrying out concurrent measurement of neuronal activity and CBF changes used psilocin at a dose of 2 mg/kg for pharmacological manipulation.

**Fig. 1:**
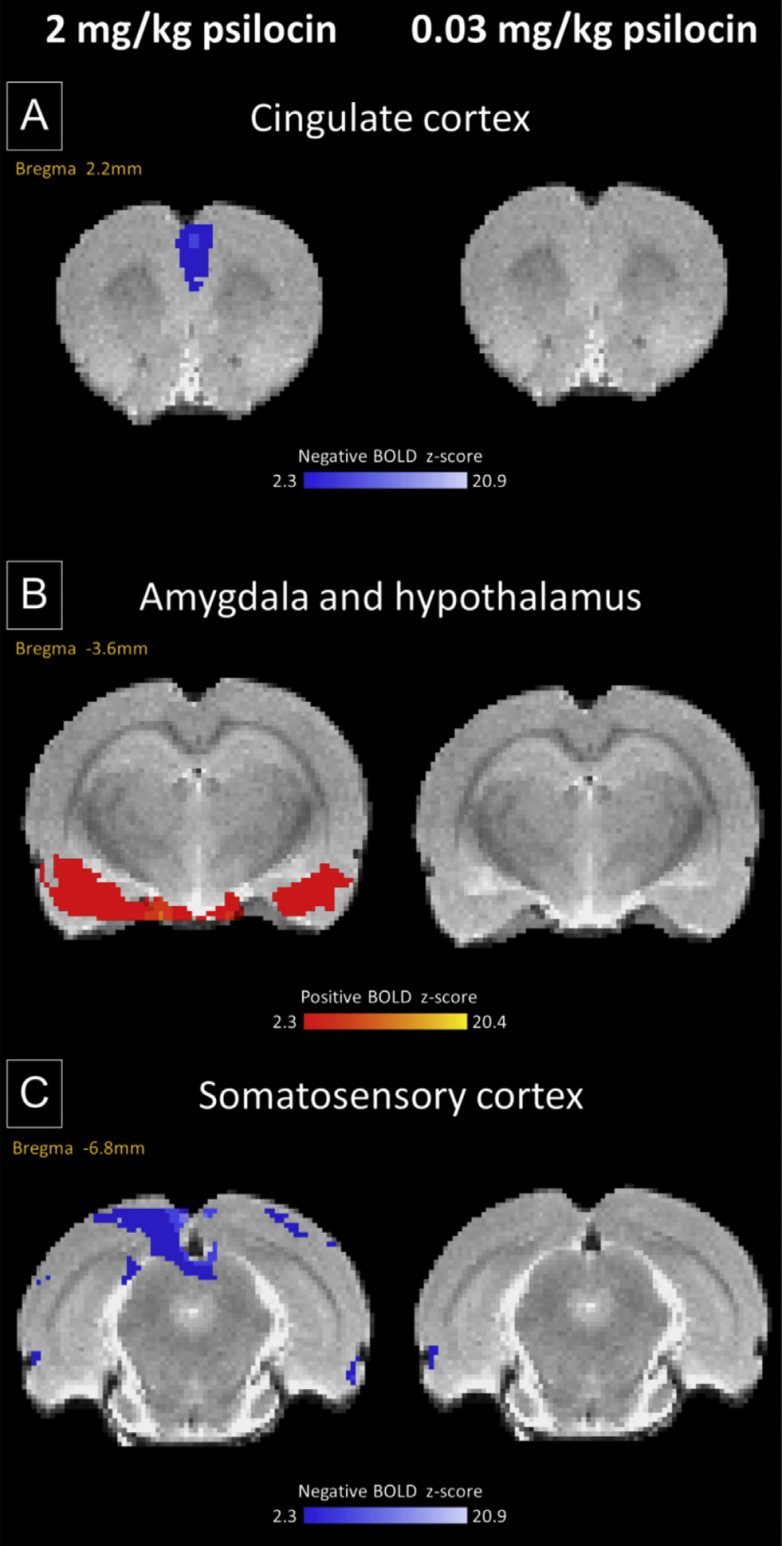
At a dose of 2 mg/kg psilocin produced BOLD signal decreases in the cingulate cortex compared to vehicle treated controls while a dose of 0.03 mg/kg produced no change (A). Signal decreases were also observed at a dose of 2 mg/kg in the somatosensory cortex (C). The same dose produced signal increases in the amygdala and hypothalamus while a dose of 0.03 mg/kg produced no change in the same regions (B). All experimental groups have N = 6.

**Fig. 2:**
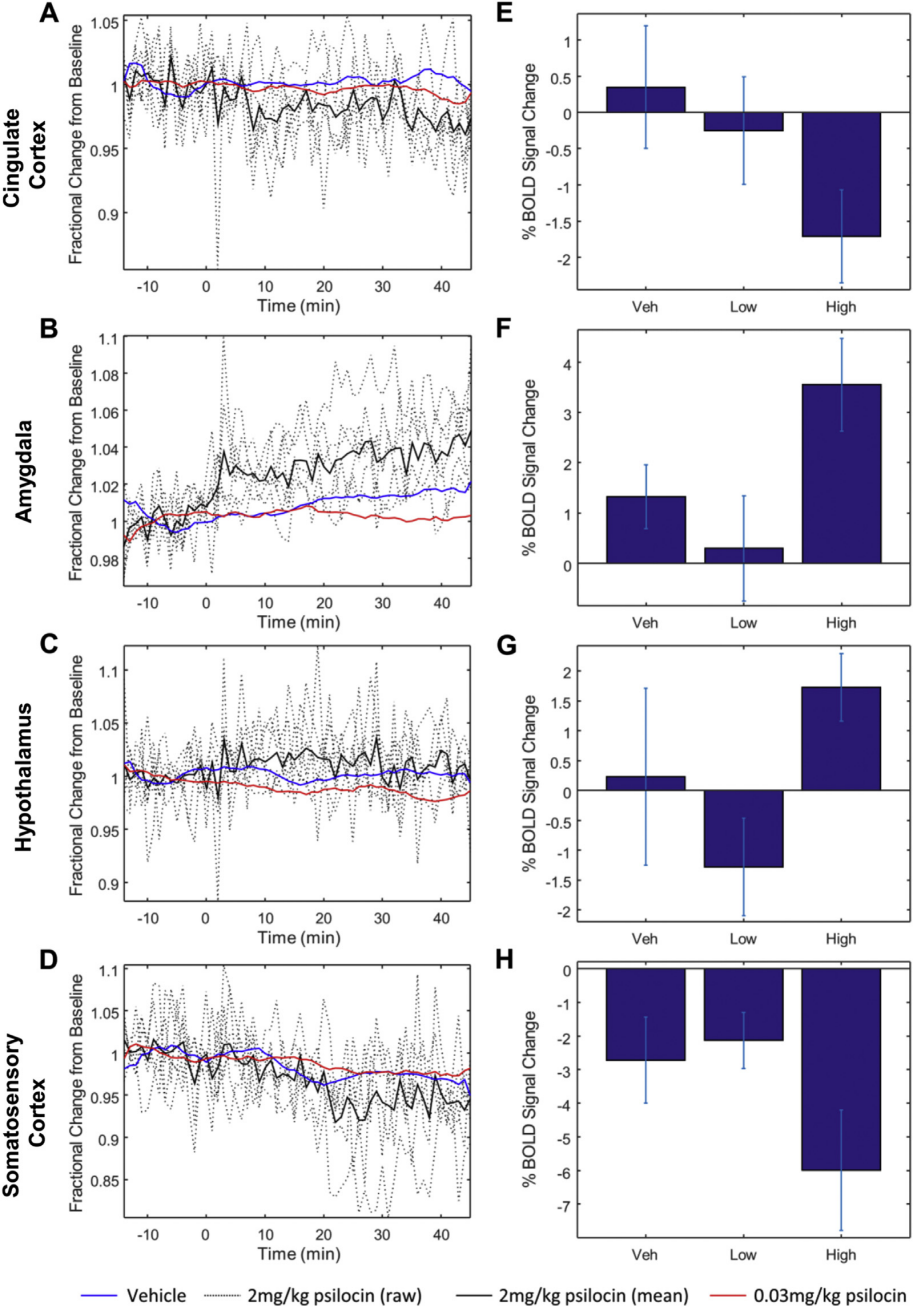
Changes in BOLD signal magnitude in brain regions identified in Fig. 1. (A–D) Time series changes in the four brain regions showing BOLD activation/deactivation. Dotted lines represent raw traces from individual animals that received a 2 mg/kg dose of psilocin, solid line is the mean of these animals. Blue and red lines represent the mean signal changes from animals that received vehicle or 0.03 mg/kg psilocin, respectively, for comparison. The average traces for these groups have been low pass filtered to improve clarity. (E–H) Percentage change in BOLD signal magnitude relative to baseline for each of the four identified brain regions. The signal magnitude was calculated by averaging over the period from 20 to 35 min after administration of 2 mg/kg psilocin (amygdala, cingulate cortex and somatosensory cortex) or 5–20 min after drug administration (hypothalamus). Error bars represent standard error of the mean. MANOVA performed on the signal magnitude values (as summarised in E–H) revealed a significant effect of drug dose (Hotelling's Trace: df = 8, 22; F = 2.424; p = 0.048). (For interpretation of the references to colour in this figure legend, the reader is referred to the web version of this article.)

Time series extracted from regions showing significant BOLD signal changes demonstrated decreases in BOLD signal in the cingulate cortex and somatosensory cortex after drug administration in animals receiving 2 mg/kg psilocin (Fig. 1, Fig. 2A, E, D & H), but an increased BOLD signal in the hypothalamus and amygdala (Fig. 1, Fig. 2B, F, C & G). Whole brain activation maps for animals that received psilocin at a dose of 2 mg/kg are shown in Supplementary Figs. 1 and 2. A NIFTI format raw brain image from a representative animal in the vehicle control group is also provided in the Supplementary materials for this paper.

### 3.2 Haemodynamic responses to somatosensory stimulation

**Fig. 3:**
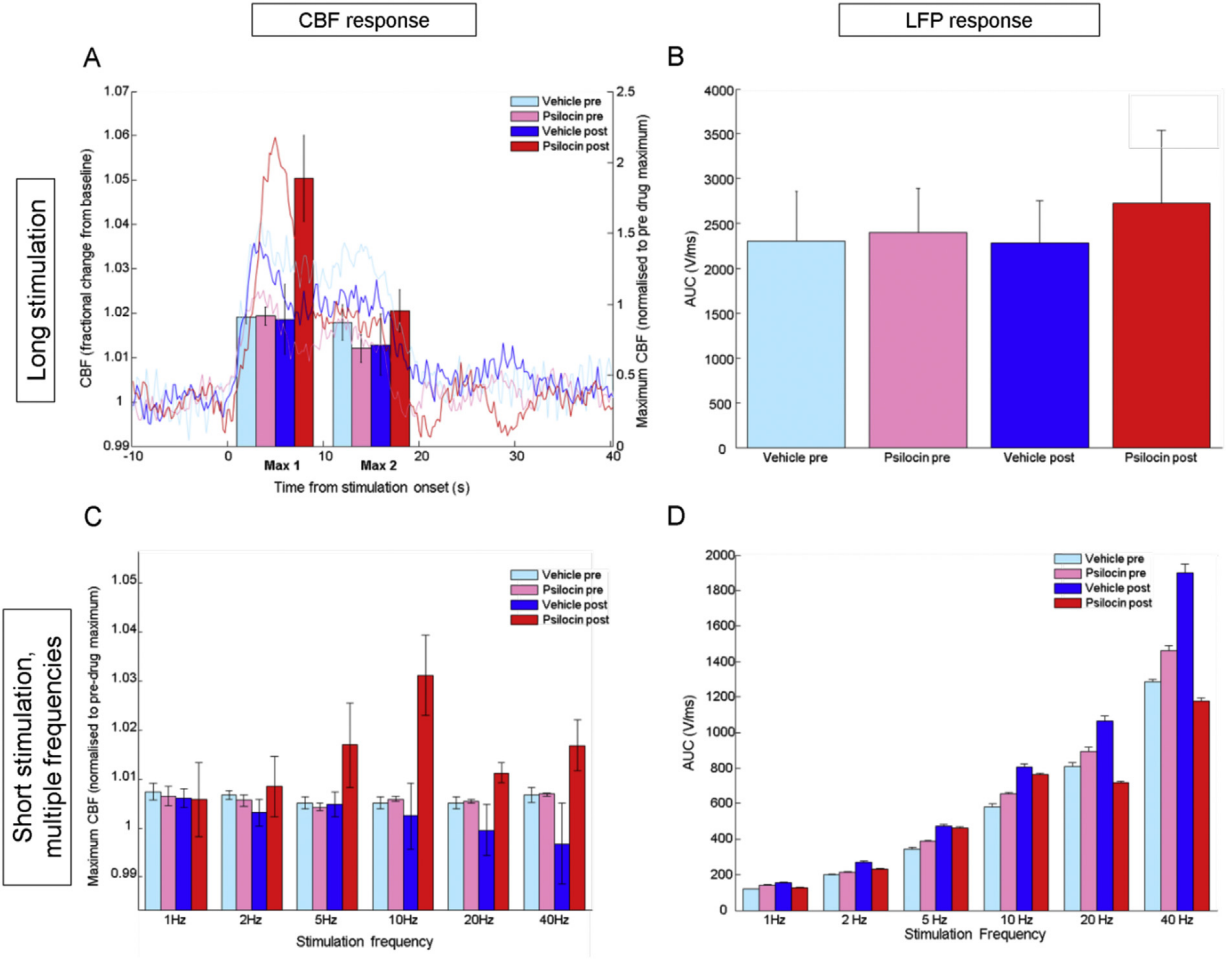
Treatment with 2 mg/kg psilocin increases the magnitude of the haemodynamic response to whisker stimulation in the somatosensory cortex. In a long stimulation paradigm this was evident in the initial phase of the response where animals that received psilocin had an increased response amplitude (A). The magnitude of the neuronal response in the somatosensory cortex to the same stimulation paradigm was unaltered (B). When shorter stimulations with multiple frequencies were applied this effect was significant only at 10 Hz (1C). Neuronal response magnitude was decreased after psilocin administration at high stimulation frequencies in the short stimulation, mixed frequency paradigm (D). Data are mean ± SEM, N = 6 for psilocin treatment, N = 5 for vehicle treatment.

Psilocin treated animals showed increased haemodynamic response amplitude to whisker stimulation following drug administration (Fig. 3A). There was a significant effect of drug treatment on the amplitude of the initial (0–8 s from stimulation onset) part of the CBF response to long, 16 s duration, 10 Hz stimulation and a significant interaction between this effect and pre-/post-drug administration. There was a significant effect of pre-/post-drug administration on this measure (statistics summarised in Table 2). There were no significant effects on the maximum of the second part of the response (10–20 s from stimulation onset; statistics summarised in Table 2).

Maximum CBF responses to mixed frequency (1–40 Hz) whisker stimulations of short, 2 s duration did not differ according to drug treatment or pre-/post-drug administration and no significant effect of whisker stimulation frequency on response maxima was found (Fig. 3C, statistics summarised in Table 3). In order to investigate whether the effects observed with a longer (16 s duration) stimulation (see above) were replicated for this specific frequency (10 Hz) in the short stimulation condition, a separate two-way ANOVA was run using only data for the 2 s, 10 Hz stimuli. This showed a significant effect of drug treatment and a significant interaction effect of drug treatment with pre-/post-drug administration on maximum CBF response to whisker stimulation (Table 3, bottom row). Specifically, psilocin treated animals showed a greater maximum CBF response after receiving drug treatment (Fig. 3C) in agreement with the data from the 16 s stimulation condition. Results of equivalent analyses for the other five stimulation frequencies and statistics are shown in Supplementary Table S1.

No effects of drug treatment were evident on the latency of the haemodynamic responses to either long, 16 s duration, or short mixed-frequency whisker stimulation (statistics shown in Table 2). There were no significant differences between the maximal CBF responses of vehicle and psilocin treated animals to 30 s of hypercapnia (statistics shown in Table 4).

### 3.3 Neuronal responses to somatosensory stimulation

**Fig. 4:**
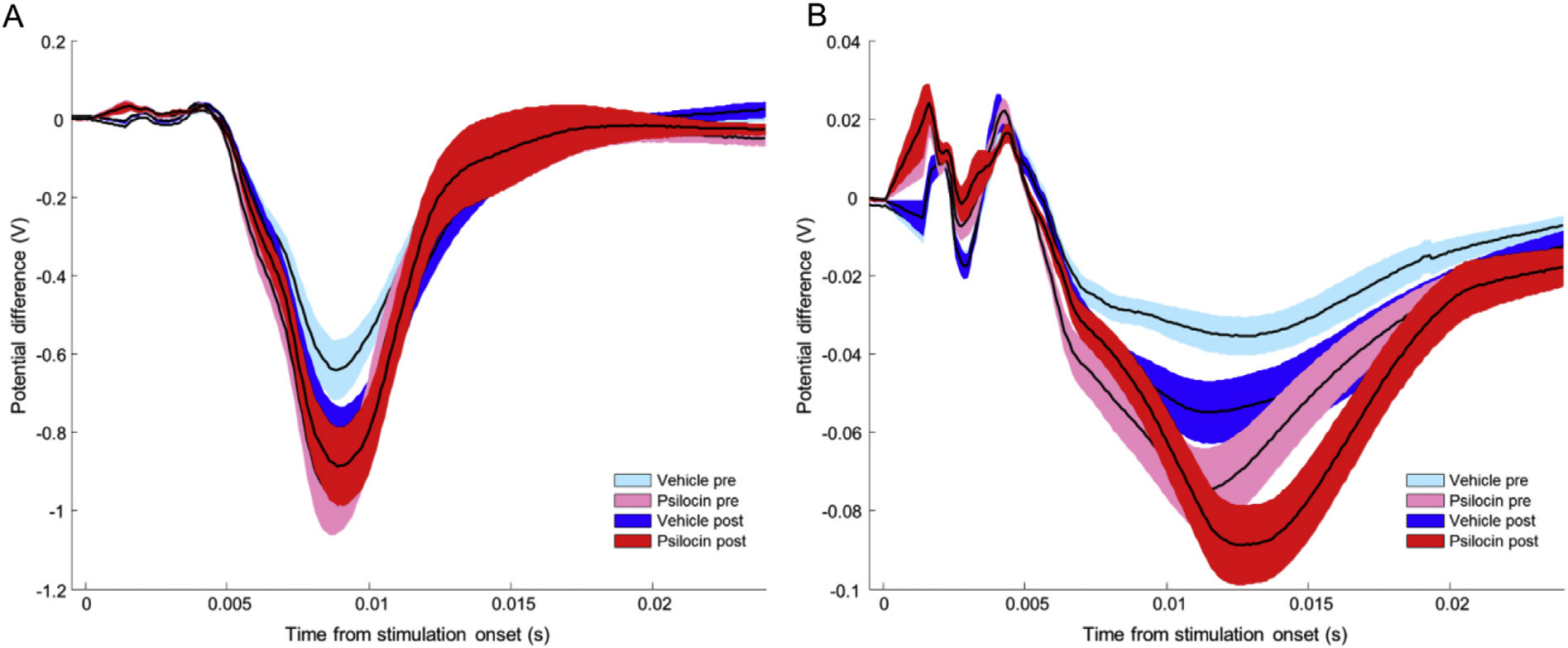
Average neuronal responses to long, 16 s duration, 10 Hz whisker stimulation were not significantly altered by administration of psilocin. Black lines show response means, coloured bands represent SEM, N = 6 for psilocin treatment, N = 5 for vehicle treatment. (A) The average response to the first pulse in the stimulation train. (B) The average response to all subsequent pulses in the train.

Neuronal responses in the long, 16 s whisker stimulation condition, as determined by the AUC of LFP responses, were not significantly altered by drug treatment condition (see Fig. 3, Fig. 4; statistics summarised in Table 5).

In contrast, in the short, mixed frequency stimulation condition, psilocin treated animals showed a significant reduction in neuronal response magnitude (Fig. 3D). The drug type × pre-/post-drug administration × stimulation frequency interaction effect was significant. There was also a significant interaction between drug type and pre-/post-drug administration as well as an effect of drug type. There were also significant effects of pre-/post-drug administration, stimulation frequency and a significant interaction between these effects. *Post-hoc* a two-way ANOVA was carried out on each stimulation frequency to determine whether the effects of psilocin administration were observed at specific frequencies. The Bonferroni technique was used to adjust the α level for multiple comparisons (i.e. *p* < 0.0083 was considered significant). A significant drug type × pre-/post-drug administration effect was found at all frequencies with psilocin treated animals showing a decrease in AUC of the LFP response after drug administration. This effect was most pronounced at 20 and 40 Hz stimulation frequencies (Fig. 3D; statistics summarised in Table 6).

## 4 Discussion

We have demonstrated that the BOLD signal changes observed in response to psilocin administration are associated with alterations in neurovascular coupling. Psilocin produced BOLD signal changes in anatomically distinct regions including olfactory and limbic systems, visual system, hippocampus, hypothalamus, prelimbic, cingulate and somatosensory cortices at a dose of 2 mg/kg. BOLD signal changes did not occur in the same direction throughout the brain, with signal increases and decreases observed in different regions. Detailed investigation of CBF and neuronal responses to whisker stimulation in the somatosensory cortex, a region of psilocin-mediated decreased phMRI signal, demonstrated stimulation frequency specific augmentation of the CBF response by psilocin, accompanied by decreased neuronal response magnitudes; indicating modulation of the relationship between neuronal activity and CBF. These findings suggest that psilocin alters neurovascular coupling and that caution is required when making inferences about drug effects on neuronal activity from changes detected in neuroimaging signals.

The opposing directionality of LFP and CBF response changes in the somatosensory cortex due to psilocin suggest altered neurovascular coupling is partly responsible for the observed BOLD-phMRI changes. The unaltered CBF responses to hypercapnia challenge indicate that vascular reactivity was unaffected by administration of psilocin (Rostrup et al., 1994). 5-HT_2A_ receptors are also expressed on both pyramidal cells (Willins et al., 1997) and inhibitory interneurons (Andrade, 2011, Puig and Gulledge, 2011) suggesting that agonism of this receptor can have both excitatory and inhibitory effects on downstream neuronal signalling. Cortical inhibitory interneurons are capable of producing both vasodilation and vasoconstriction (Cauli et al., 2004), providing a mechanism by which reduced synaptic activity may actually be associated with increased CBF. The linearity of the neurovascular response to whisker stimulation has also been proposed to be dependent on changes in the background balance of inhibition and excitation in the brain (Buzsáki et al., 2007, Lauritzen et al., 2012), providing additional means by which neurovascular coupling may be altered by psilocin. The role of extra-cortical effects of psilocin on other receptor subtypes in modulating the somatosensory response to stimulation cannot be ruled out. For instance, the locus coeruleus (LC) modulates CBF responses to whisker stimulation (Toussay et al., 2013) and LC activity is known to be inhibited by 2,5-dimethoxy-4-iodoamphetamine (DOI), another non-selective 5-HT_2A_ receptor agonist (Szabo and Blier, 2001). In addition, Fig. 3 shows that neuronal response to whisker stimulation at 40 Hz increased over time in vehicle-treated controls. The directionality of this effect is opposite to that found in the psilocin group. We are unsure as to why this occurred, but it could be due to an effect of anaesthetic depth varying over time or the effects of the vehicle, tartaric acid. Since the change is in the opposite direction to that induced by psilocin, it is possible that the changes we observed in the psilocin group are being partially masked by this effect.

Recent work by Riga et al. (2014) investigated the neuronal and fMRI signal effects of the 5-HT_2A_ receptor agonist and hallucinogen 5-Methoxy-N,N-dimethyltryptamine (5-MeO-DMT), also in rats. In agreement with the present findings they report a mixed pattern of positive and negative BOLD signal changes in the brain, with widespread areas of decreased BOLD signal occurring in sensory cortex and subcortical BOLD signal increases (e.g. in hippocampus). Riga et al. also showed reduced low frequency (0.15–4 Hz) oscillations and increased pyramidal spiking activity associated with decreased BOLD signal in medial prefrontal cortex following 5-MeO-DMT administration. Against a background of reduced neuronal oscillatory power in this ‘delta’ range, haemodynamic response signals have been shown elsewhere to increase (Niessing et al., 2005), providing an additional explanation for the enhancement of stimulus-evoked haemodynamic responses observed here following psilocin treatment.

Psilocin has both on- and off-target actions that may be important for the interpretation of the results reported here. Although there is strong evidence that the hallucinogenic effects of psilocin are associated with 5-HT_2A_ receptor activation (Quednow et al., 2012, Halberstadt, 2015), the indirect nature of using haemodynamic neuroimaging signals as proxy measures for neuronal activity make off-target effects, including non-hallucinogenic and peripheral actions mediated by other receptor subtypes, an important consideration. For example, psilocin has effects at most 5-HT receptor subtypes and also some dopamine receptors (e.g. see Ray, 2010). In particular, the effects of psilocin on 5-HT_1B/1D_ receptors, which are expressed on cerebral vasculature, may have a direct ‘non-neuronal’ role in mediating haemodynamics. These off-target effects could contribute to the changes in neural-haemodynamic coupling observed here, and would again limit the extent to which pharmacological neuroimaging readouts could be interpreted as indicating increases or decreases in neuronal activity.

The findings presented here are particularly relevant to situations where alterations in brain responses to sensory (e.g. visual, auditory) stimulation are being investigated using neuroimaging, as they demonstrate that serotonergic manipulations may alter the relationship of BOLD signal changes to neuronal activity. To exemplify, in isolation, our haemodynamic (i.e. neuroimaging) data suggests that psilocin *increases* the magnitude of cortical responses to sensory stimulation (Fig. 3A). In fact, our concurrent measurement of neuronal response magnitudes suggests that if anything, the opposite is true. Furthermore, these findings may have implications for neuroimaging studies in clinical cohorts where serotonergic anomalies are known to exist (Rive et al., 2013). Alterations of BOLD responses to stimulation in conjunction with pharmacological serotonin manipulations, such as with selective serotonin reuptake inhibitors (SSRIs), have been documented in human subjects (Klomp et al., 2013, Rive et al., 2013, Windischberger et al., 2010) and our findings, based on concurrent measurement of neuronal and CBF changes underlying BOLD signals, demonstrate why caution should be exercised in the interpretation of such findings. Specifically, many interpretations of BOLD-fMRI signal changes assume a monotonic relationship between evoked neuronal and haemodynamic response magnitudes, something we have here demonstrated to be altered by psilocin administration. Furthermore, the alteration in neurovascular coupling we report here might explain, in part, the apparent discrepancy between fMRI and PET findings of decreased CBF (Carhart-Harris et al., 2012a) and increased glucose metabolism (Gouzoulis-Mayfrank et al., 1999, Vollenweider et al., 1997) in human studies with psilocybin and related drugs. Although it is widely assumed that neuronal activity, glucose metabolism, cerebral blood flow and fMRI BOLD signals increase (or decrease) in tandem, this assumption has only been extensively tested in conditions of sensory stimulus-induced activation change (and where caveats have been found): physiological investigations in preclinical models will continue to be important for detailing these relationships in pharmacological neuroimaging contexts.

We have shown that the anatomical areas affected by psilocin in the rat are congruent with those affected in the human brain (Carhart-Harris et al., 2012a). The direction of our observed changes in the rat is not, however, entirely consistent with those observed in humans. In humans, BOLD signals were uniformly decreased in response to psilocin administration, while BOLD signals in rat were observed to increase as well as decrease, depending on the anatomical location. The divergence in direction of signal change may reflect either an interspecies difference in the effects of the drug or the larger effective dose used in our study compared to that of Carhart-Harris and colleagues (Carhart-Harris et al., 2012a) (2 mg/kg vs. 2 mg/total body weight, respectively). The absence of changes observed at 0.03 mg/kg in our study may be attributable to interspecies differences between rats and humans. The dose of 0.03 mg/kg was calculated to be equivalent to the dose used by Carhart-Harris and colleagues (2 mg/subject in their study divided by our assumed 70 kg bodyweight per subject). Without scaling for interspecies pharmacokinetic differences, however, there may not be direct concordance between the two doses (Boxenbaum, 1982, Lin, 1998).

Another important difference between our work and previous human studies is the use of anaesthesia. Although methods to acquire neuronal and haemodynamic data in awake animals have been developed in our laboratory and elsewhere (Martin et al., 2013, Martin et al., 2013, Takuwa et al., 2009), anaesthesia remains important to enable invasive and concurrent measurement of neuronal and haemodynamic data, especially in the context of drug treatments like psilocin which are well-known for eliciting head-twitch responses (Corne and Pickering, 1967). Anaesthetics, including isoflurane as used in this study (the most popular choice for preclinical phMRI research (Haensel et al., 2015)), are known modulators of haemodynamic responses (Austin et al., 2005, Martin et al., 2006, Tsurugizawa et al., 2010). Our experimental design, which included within-subjects measurement of the effects of psilocin, will to some extent mitigate against this as a confounding factor as animal anaesthesia levels were constant throughout the pre- and post-drug conditions. The observation of CBF responses to stimulation under anaesthesia even in vehicle treated control animals as well as prior to drug administration also suggests that this effect did not obscure CBF responses in this study. This is consistent with data from previous work (Franceschini et al., 2010, Masamoto et al., 2009, Sicard and Duong, 2005), which shows that under isoflurane anaesthesia, capacity for blood flow changes in response to neuronal activation remains. The effects of isoflurane on neurovascular coupling have also been demonstrated to be comparable to those of other anaesthetics (Franceschini et al., 2010). As a result we are confident that the effects of isoflurane on the results obtained in this study are small and were further minimised by using the lowest necessary dose of isoflurane for anaesthesia during experimental manipulations. Isoflurane is also known to have a variety of neuronal effects (Haensel et al., 2015), among these, it is known to increase permeability of GABA_A_ receptors to chloride. This effect could be relevant to the current study given the location of 5-HT_2A_ receptors on GABAergic neurons, however we did not find psilocin related alterations in the neuronal responses to whisker stimulation suggesting that any such effects were minimal. It has been suggested that the sedative dexmedetomidine could provide an alternate, ‘compromise’ means of avoiding the challenges of both general anaesthesia and awake rodent studies (Weber et al., 2006). However, recent research has demonstrated this drug to have a range of vascular, neuronal and neurovascular actions (Fukuda et al., 2013) and as such was judged to be less suitable for the present study.

In conclusion, psilocin induced alterations in neurovascular coupling have implications for the interpretation of BOLD-phMRI based investigation of psilocin, and possibly other 5-HT agonists, as well as underlining the general need for caution in interpreting fMRI studies in the context of neuropharmacological manipulations (Shih et al., 2009). Determining a plausible mechanism of action for this effect is necessary to progress research using such drugs. phMRI studies in human subjects are important in this endeavour, but additional studies will be necessary to connect the haemodynamic readouts provided by such non-invasive neuroimaging methods to better validated makers of neuronal activity, such as those provided by experimental animal studies. More generally, we suggest that such research is increasingly needed to underpin interpretation of non-invasive (haemodynamic) functional imaging studies of drug action in the human brain.

## Figures and Tables

**Table 1:** Anatomical regions showing BOLD signal changes following administration of 2 mg/kg psilocin.

Increased BOLD signal vs vehicle	Decreased BOLD signal vs vehicle
Olfactory and limbic areas	Prelimbic cortex
*Amygdalar nuclei*	Cingulate cortex
*Olfactory tubercle*	Primary motor cortex
*Piriform cortex*	Secondary motor cortex
*Endopiriform nucleus*	Somatosensory cortex
Substantia nigra, pars reticulata	*Barrel cortex*
Optic tract	Thalamic nuclei
Hypothalamic nuclei	*Dorsal lateral geniculate nucleus*
Hippocampus	*Lateral posterior thalamic nucleus*
*Dentate gyrus*	Dorsolateral periaqueductal grey
*Subiculum*	Superior colliculus
*Presubiculum*	Entorhinal cortex
Cerebral peduncle	Hippocampus
Pontine nuclei	*CA3*
*Gigantocellular reticular nucleus*	*CA1*
*Paramedian reticular nucleus*	*Fimbria*
	Lateral and medial habenulae
	Visual cortex

**Table 2:** Summary of statistical analyses of haemodynamic responses to 10 Hz, 16 s duration stimulation. * = *p* < 0.05.

Parameter	Factor	df	F	*p*
Maximum of first phase (0–8 s from stimulation onset) of CBF response to 10 Hz, 16 s duration stimulation	Drug treatment	1,9	5.338	0.046*
Pre/post drug administration	1,9	6.632	0.030*
Drug treatment × Pre/post drug administration interaction	1,9	6.187	0.035*
Maximum of second phase (10–20 s from stimulation onset) of CBF response to 10 Hz, 16 s duration stimulation	Drug treatment	1,9	0.033	0.861
Pre/post drug administration	1,9	0.137	0.720
Drug treatment × Pre/post drug administration interaction	1,9	2.037	0.187
Time to half maximum CBF response to 10 Hz, 16 s duration stimulation	Drug treatment	1,9	0.198	0.667
Pre/post drug administration	1,9	0.031	0.863
Drug treatment × Pre/post drug administration interaction	1,9	0.131	0.726

**Table 3:** Summary of statistical analyses of haemodynamic responses to mixed frequency, 2 s duration stimulation.

Parameter	Factor	df	F	*p*
Maximum CBF response to mixed frequency, 2 s duration stimulation	Drug treatment	1,9	2.787	0.129
Pre/post drug administration	1,9	0.524	0.488
Drug treatment × Pre/post drug administration interaction	1,9	3.110	0.112
Stimulation frequency	5,45	1.207	0.321
Stimulation frequency × drug treatment interaction	5,45	2.115	0.081
Pre/post drug administration × frequency interaction	5,45	1.965	1.02
Pre/post drug administration × frequency × drug treatment interaction	5,45	1.866	0.119

Time to half maximum CBF response to mixed frequency, 2 s duration stimulation	Drug treatment	1,9	1.314	0.281
Pre/post drug administration	1,9	1.029	0.337
Drug treatment × Pre/post drug administration interaction	1,9	1.200	0.302
Stimulation frequency	5,45	1.176	0.336
Stimulation frequency × drug treatment interaction	5,45	0.723	0.610
Pre/post drug administration × frequency interaction	5,45	1.498	0.209
	Pre/post drug administration × frequency × drug treatment interaction	5,45	1.800	0.132

Analysis of 10 Hz CBF response data only	Drug treatment	1,9	5.462	0.044*
Pre/post drug administration	1,9	3.359	0.100
Drug treatment × Pre/post drug administration interaction	1,9	5.125	0.050*

*=p<0.05.

**Table 4:** Summary of statistical analyses of haemodynamic responses to 30 s increased CO_2_ concentration.

Parameter	Factor	df	F	*p*
Maximum CBF response to 30 s increased CO_2_ concentration	Drug treatment	1,9	2.754	0.131
Pre/post drug administration	1,9	2.805	0.128
Drug treatment × Pre/post drug administration interaction	1,9	2.940	0.121

**Table 5:** Summary of statistical analyses of neuronal response magnitudes to 10 Hz, 16 s duration stimulation.

Stimulation frequency	Factor	df	F	*p*
10 Hz, 16 s stimulation	Drug treatment	1,9	0.117	0.740
Pre/post drug administration	1,9	0.136	0.721
Drug treatment × Pre/post drug administration interaction	1,9	0.172	0.688

**Table 6:** Summary of statistical analyses of neuronal response magnitudes to mixed frequency, 2 s duration stimulation. * = *p* < 0.0083.

Stimulation frequency	Factor	df	F	*p*
Mixed frequency, 2 s stimulation	Drug treatment	1,9	30.539	<0.001*
Pre/post drug administration	1,9	48.122	<0.001*
Drug treatment × Pre/post drug administration interaction	1,9	111.839	<0.001*
Stimulation frequency	1,9	3631.889	<0.001*
Stimulation frequency × drug treatment interaction	5,45	50.504	<0.001*
Pre/post drug administration × frequency interaction	5,45	21.432	<0.001*
Pre/post drug administration × frequency × drug treatment interaction	5,45	136.091	<0.001*

1 Hz	Drug treatment	1,9	2.009	0.187
Pre/post drug administration	1,9	15.051	0.003*
Drug treatment × Pre/post drug administration interaction	1,9	44.132	<0.001*

2 Hz	Drug treatment	1,9	1.983	0.189
Pre/post drug administration	1,9	80.495	<0.001*
Drug treatment × Pre/post drug administration interaction	1,9	39.832	<0.001*

5 Hz	Drug treatment	1,9	2.556	0.141
Pre/post drug administration	1,9	157.101	<0.001*
Drug treatment × Pre/post drug administration interaction	1,9	14.051	0.004*

10 Hz	Drug treatment	1,9	0.458	0.514
Pre/post drug administration	1,9	256.354	<0.001*
Drug treatment × Pre/post drug administration interaction	1,9	33.898	<0.001*

20 Hz	Drug treatment	1,9	37.478	<0.001*
Pre/post drug administration	1,9	3.610	0.087
Drug treatment × Pre/post drug administration interaction	1,9	88.602	<0.001*

40 Hz	Drug treatment	1,9	69.494	<0.001*
Pre/post drug administration	1,9	29.415	<0.001*
Drug treatment × Pre/post drug administration interaction	1,9	192.861	<0.001*

## References

[bib1] Andrade R. (2011). Serotonergic regulation of neuronal excitability in the prefrontal cortex. Neuropharmacology.

[bib2] Angenstein F., Kammerer E., Scheich H. (2009). The BOLD response in the rat hippocampus depends rather on local processing of signals than on the input or output activity. A combined functional MRI and electrophysiological study. J. Neurosci..

[bib3] Austin V.C., Blamire A.M., Allers K.A., Sharp T., Styles P., Matthews P.M., Sibson N.R. (2005). Confounding effects of anesthesia on functional activation in rodent brain: a study of halothane and alpha-chloralose anesthesia. Neuroimage.

[bib4] Boorman L., Kennerley A.J., Johnston D., Jones M., Zheng Y., Redgrave P., Berwick J. (2010). Negative blood oxygen level dependence in the rat: a model for investigating the role of suppression in neurovascular coupling. J. Neurosci..

[bib5] Borsook D., Becerra L., Hargreaves R. (2006). A role for fMRI in optimizing CNS drug development. Nat. Rev. Drug Discov..

[bib6] Boxenbaum H. (1982). Interspecies scaling, allometry, physiological time, and the ground plan of pharmacokinetics. J. Pharmacokinet. Biopharm..

[bib7] Buzsáki G., Kaila K., Raichle M. (2007). Inhibition and brain work. Neuron.

[bib8] Carhart-Harris R.L., Erritzoe D., Williams T., Stone J.M., Reed L.J., Colasanti A., Tyacke R.J., Leech R., Malizia A.L., Murphy K., Hobden P., Evans J., Feilding A., Wise R.G., Nutt D.J. (2012). Neural correlates of the psychedelic state as determined by fMRI studies with psilocybin. Proc. Natl. Acad. Sci. U. S. A..

[bib9] Carhart-Harris R.L., Leech R., Williams T.M., Erritzoe D., Abbasi N., Bargiotas T., Hobden P., Sharp D.J., Evans J., Feilding A., Wise R.G., Nutt D.J. (2012). Implications for psychedelic-assisted psychotherapy: functional magnetic resonance imaging study with psilocybin. Br. J. Psychiatry.

[bib10] Cauli B., Tong X.-K., Rancillac A., Serluca N., Lambolez B., Rossier J., Hamel E. (2004). Cortical GABA interneurons in neurovascular coupling: relays for subcortical vasoactive pathways. J. Neurosci..

[bib11] Cohen Z., Bonvento G., Lacombe P., Hamel E. (1996). Serotonin in the regulation of brain microcirculation. Prog. Neurobiol..

[bib12] Corne S.J., Pickering R.W. (1967). A possible correlation between drug-induced hallucinations in man and a behavioural response in mice. Psychopharmacologia.

[bib13] Couch Y., Martin C.J., Howarth C., Raley J., Khrapitchev A.A., Stratford M., Sharp T., Sibson N.R., Anthony D.C. (2013). Systemic inflammation alters central 5-HT function as determined by pharmacological MRI. Neuroimage.

[bib14] Devor A., Ulbert I., Dunn A.K., Narayanan S.N., Jones S.R., Andermann M.L., Boas D.A., Dale A.M. (2005). Coupling of the cortical hemodynamic response to cortical and thalamic neuronal activity. Proc. Natl. Acad. Sci. U. S. A..

[bib15] Fleisher A.S., Sherzai A., Taylor C., Langbaum J.B.S., Chen K., Buxton R.B. (2009). Resting-state BOLD networks versus task-associated functional MRI for distinguishing Alzheimer's disease risk groups. Neuroimage.

[bib16] Franceschini M.A., Radhakrishnan H., Thakur K., Wu W., Ruvinskaya S., Carp S., Boas D.A. (2010). The effect of different anesthetics on neurovascular coupling. Neuroimage.

[bib17] Fukuda M., Suzuki N., Maruyama S., Dobashi K., Kitamura A., Sakai F. (2002). Effects of sumatriptan on cerebral blood flow under normo- and hypercapnia in rats. Cephalalgia.

[bib18] Fukuda M., Vazquez A.L., Zong X., Kim S.-G. (2013). Effects of the α2-adrenergic receptor agonist dexmedetomidine on neural, vascular and BOLD fMRI responses in the somatosensory cortex. Eur. J. Neurosci..

[bib19] Gouzoulis-Mayfrank E., Schreckenberger M., Sabri O., Arning C., Thelen B., Spitzer M., Kovar K.A., Hermle L., Büll U., Sass H. (1999). Neurometabolic effects of psilocybin, 3,4-methylenedioxyethylamphetamine (MDE) and d-methamphetamine in healthy volunteers. A double-blind, placebo-controlled PET study with [18F]FDG. Neuropsychopharmacology.

[bib20] Grob C.S., Danforth A.L., Chopra G.S., Hagerty M., McKay C.R., Halberstadt A.L., Greer G.R. (2011). Pilot study of psilocybin treatment for anxiety in patients with advanced-stage cancer. Arch. Gen. Psychiatry.

[bib21] Gupta S., Villalón C.M. (2010). The relevance of preclinical research models for the development of antimigraine drugs: focus on 5-HT(1B/1D) and CGRP receptors. Pharmacol. Ther..

[bib22] Haensel J., Spain A., Martin C. (2015). A systematic review of physiological methods in rodent pharmacological MRI studies. Psychopharmacology (Berl).

[bib23] Halberstadt A.L., Geyer M.A. (2011). Multiple receptors contribute to the behavioral effects of indoleamine hallucinogens. Neuropharmacology.

[bib24] Halberstadt A.L. (2015). Recent advances in the neuropsychopharmacology of serotonergic hallucinogens. Behav. Brain Res..

[bib25] Hamel E. (2006). Perivascular nerves and the regulation of cerebrovascular tone. J. Appl. Physiol..

[bib26] Harel N., Lee S.-P., Nagaoka T., Kim D.-S., Kim S.-G. (2002). Origin of negative blood oxygenation level-dependent fMRI signals. J. Cereb. Blood Flow Metab..

[bib27] Hasler F., Bourquin D., Brenneisen R., Bär T., Vollenweider F.X. (1997). Determination of psilocin and 4-hydroxyindole-3-acetic acid in plasma by HPLC-ECD and pharmacokinetic profiles of oral and intravenous psilocybin in man. Pharm. Acta Helv..

[bib28] Jenkinson M., Beckmann C.F., Behrens T.E.J., Woolrich M.W., Smith S.M. (2012). FSL. Neuroimage.

[bib29] Kim S.-G., Ogawa S. (2012). Biophysical and physiological origins of blood oxygenation level-dependent fMRI signals. J. Cereb. Blood Flow Metab..

[bib30] Klomp A., van Wingen G.A., de Ruiter M.B., Caan M.W.A., Denys D., Reneman L. (2013). Test-retest reliability of task-related pharmacological MRI with a single-dose oral citalopram challenge. Neuroimage.

[bib31] Kovács A., Hársing L.G., Szénási G. (2012). Vasoconstrictor 5-HT receptors in the smooth muscle of the rat middle cerebral artery. Eur. J. Pharmacol..

[bib32] Lauritzen M., Mathiesen C., Schaefer K., Thomsen K.J. (2012). Neuronal inhibition and excitation, and the dichotomic control of brain hemodynamic and oxygen responses. Neuroimage.

[bib33] Lin J.H. (1998). Applications and limitations of interspecies scaling and in vitro extrapolation in pharmacokinetics. Drug Metab. Dispos..

[bib34] Logothetis N.K. (2008). What we can do and what we cannot do with fMRI. Nature.

[bib35] Martin C., Martindale J., Berwick J., Mayhew J. (2006). Investigating neural-hemodynamic coupling and the hemodynamic response function in the awake rat. Neuroimage.

[bib36] Martin C., Sibson N.R. (2008). Pharmacological MRI in animal models: a useful tool for 5-HT research?. Neuropharmacology.

[bib37] Martin C., Zheng Y., Sibson N.R., Mayhew J.E.W., Berwick J. (2013). Complex spatiotemporal haemodynamic response following sensory stimulation in the awake rat. Neuroimage.

[bib38] Martin C.J., Kennerley A.J., Berwick J., Port M., Mayhew J.E.W. (2013). Functional MRI in conscious rats using a chronically implanted surface coil. J. Magn. Reson. Imaging.

[bib39] Martin G.R. (1994). Vascular receptors for 5-hydroxytryptamine: distribution, function and classification. Pharmacol. Ther..

[bib40] Masamoto K., Fukuda M., Vazquez A., Kim S.-G. (2009). Dose-dependent effect of isoflurane on neurovascular coupling in rat cerebral cortex. Eur. J. Neurosci..

[bib41] Mukherjee P., Whalley H.C., McKirdy J.W., Sprengelmeyer R., Young A.W., McIntosh A.M., Lawrie S.M., Hall J. (2014). Altered amygdala connectivity within the social brain in schizophrenia. Schizophr. Bull..

[bib42] Murphy S.E., Mackay C.E. (2011). Using MRI to measure drug action: caveats and new directions. J. Psychopharmacol..

[bib43] Niessing J., Ebisch B., Schmidt K.E., Niessing M., Singer W., Galuske R.A. (2005). Hemodynamic signals correlate tightly with synchronized gamma oscillations. Science.

[bib44] Passie T., Seifert J., Schneider U., Emrich H.M. (2002). The pharmacology of psilocybin. Addict. Biol..

[bib45] Puig M.V., Gulledge A.T. (2011). Serotonin and prefrontal cortex function: neurons, networks, and circuits. Mol. Neurobiol..

[bib46] Quednow B.B., Kometer M., Geyer M.A., Vollenweider F.X. (2012). Psilocybin-induced deficits in automatic and controlled inhibition are attenuated by ketanserin in healthy human volunteers. Neuropsychopharmacology.

[bib47] Ray T.S. (2010). Psychedelics and the human receptorome. PLoS One.

[bib48] Reynell C., Harris J.J. (2013). The BOLD signal and neurovascular coupling in autism. Dev. Cogn. Neurosci..

[bib49] Riga M.S., Soria G., Tudela R., Artigas F., Celada P. (2014). The natural hallucinogen 5-MeO-DMT, component of Ayahuasca, disrupts cortical function in rats: reversal by antipsychotic drugs. Int. J. Neuropsychopharmacol..

[bib50] Rive M.M., van Rooijen G., Veltman D.J., Phillips M.L., Schene A.H., Ruhé H.G. (2013). Neural correlates of dysfunctional emotion regulation in major depressive disorder. A systematic review of neuroimaging studies. Neurosci. Biobehav. Rev..

[bib51] Rostrup E., Larsson H.B., Toft P.B., Garde K., Thomsen C., Ring P., Søndergaard L., Henriksen O. (1994). Functional MRI of CO2 induced increase in cerebral perfusion. NMR Biomed..

[bib52] Schwarz A.J., Gozzi A., Reese T., Bifone A. (2007). In vivo mapping of functional connectivity in neurotransmitter systems using pharmacological MRI. Neuroimage.

[bib53] Schweinhardt P., Fransson P., Olson L., Spenger C., Andersson J.L.R. (2003). A template for spatial normalisation of MR images of the rat brain. J. Neurosci. Methods.

[bib54] Shih Y.-Y.I., Chen C.-C.V., Shyu B.-C., Lin Z.-J., Chiang Y.-C., Jaw F.-S., Chen Y.-Y., Chang C. (2009). A new scenario for negative functional magnetic resonance imaging signals: endogenous neurotransmission. J. Neurosci..

[bib55] Shmuel A., Augath M., Oeltermann A., Logothetis N.K. (2006). Negative functional MRI response correlates with decreases in neuronal activity in monkey visual area V1. Nat. Neurosci..

[bib56] Sicard K.M., Duong T.Q. (2005). Effects of hypoxia, hyperoxia, and hypercapnia on baseline and stimulus-evoked BOLD, CBF, and CMRO2 in spontaneously breathing animals. Neuroimage.

[bib57] Szabo S.T., Blier P. (2001). Functional and pharmacological characterization of the modulatory role of serotonin on the firing activity of locus coeruleus norepinephrine neurons. Brain Res..

[bib58] Takuwa H., Masamoto K., Obata T., Kanno I. (2009). Dynamic recording of ongoing neurovascular activity in awake-behaving mice. J. Cereb. Blood Flow Metab..

[bib59] Toussay X., Basu K., Lacoste B., Hamel E. (2013). Locus coeruleus stimulation recruits a broad cortical neuronal network and increases cortical perfusion. J. Neurosci..

[bib60] Tsurugizawa T., Uematsu A., Uneyama H., Torii K. (2010). Effects of isoflurane and alpha-chloralose anesthesia on BOLD fMRI responses to ingested L-glutamate in rats. Neuroscience.

[bib61] Vollenweider F.X., Kometer M. (2010). The neurobiology of psychedelic drugs: implications for the treatment of mood disorders. Nat. Rev. Neurosci..

[bib62] Vollenweider F.X., Leenders K.L., Scharfetter C., Maguire P., Stadelmann O., Angst J. (1997). Positron emission tomography and fluorodeoxyglucose studies of metabolic hyperfrontality and psychopathology in the psilocybin model of psychosis. Neuropsychopharmacology.

[bib63] Vollenweider F.X., Vollenweider-Scherpenhuyzen M.F.I., Bäbler A., Vogel H., Hell D. (1998). Psilocybin induces schizophrenia-like psychosis in humans via a serotonin-2 agonist action. Neuroreport.

[bib64] Weber R., Ramos-Cabrer P., Wiedermann D., van Camp N., Hoehn M. (2006). A fully noninvasive and robust experimental protocol for longitudinal fMRI studies in the rat. Neuroimage.

[bib65] Willins D.L., Deutch A.Y., Roth B.L. (1997). Serotonin 5-HT2A receptors are expressed on pyramidal cells and interneurons in the rat cortex. Synapse.

[bib66] Windischberger C., Lanzenberger R., Holik A., Spindelegger C., Stein P., Moser U., Gerstl F., Fink M., Moser E., Kasper S. (2010). Area-specific modulation of neural activation comparing escitalopram and citalopram revealed by pharmaco-fMRI: a randomized cross-over study. Neuroimage.

[bib67] Wise R.G., Tracey I. (2006). The role of fMRI in drug discovery. J. Magn. Reson. Imaging.

[bib68] Worsley K.J., Evans A.C., Marrett S., Neelin P. (1992). A three-dimensional statistical analysis for CBF activation studies in human brain. J. Cereb. Blood Flow Metab..

