# Supplementary material for: Neurovascular and neuroimaging effects of the hallucinogenic serotonin receptor agonist psilocin in the rat brain
Source: Neuropharmacology. 2015 Dec;99:210–20. doi: 10.1016/j.neuropharm.2015.07.018 (PMC4655865; doi:10.1016/j.neuropharm.2015.07.018)
Supplement: Supplementary file 1 [file mmc1.docx]

**Supplementary information**

**
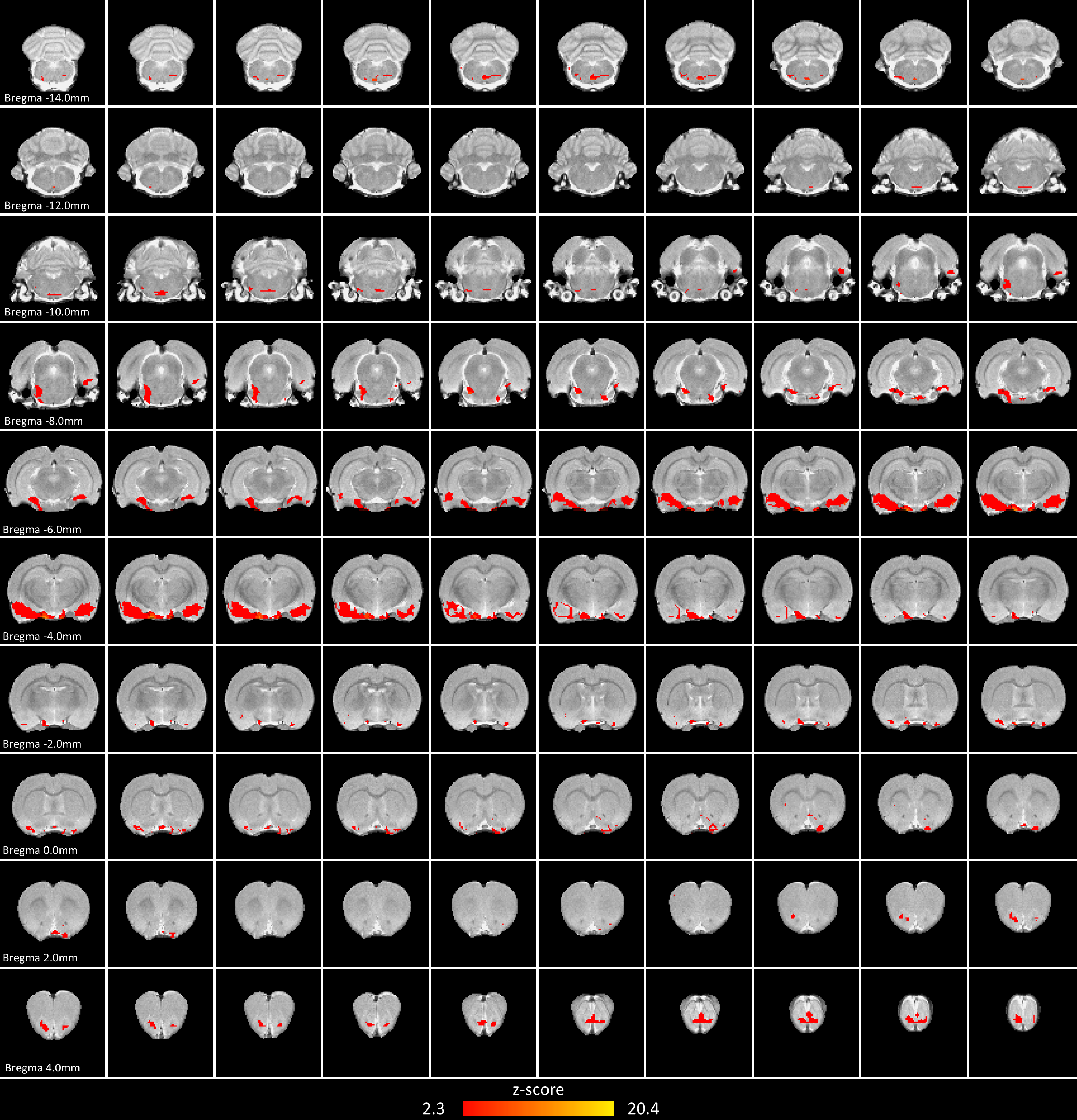
**

**Figure S1:** Whole brain maps showing BOLD signal increases in animals which received a 2mg/kg dose of psilocin compared to vehicle controls.

**
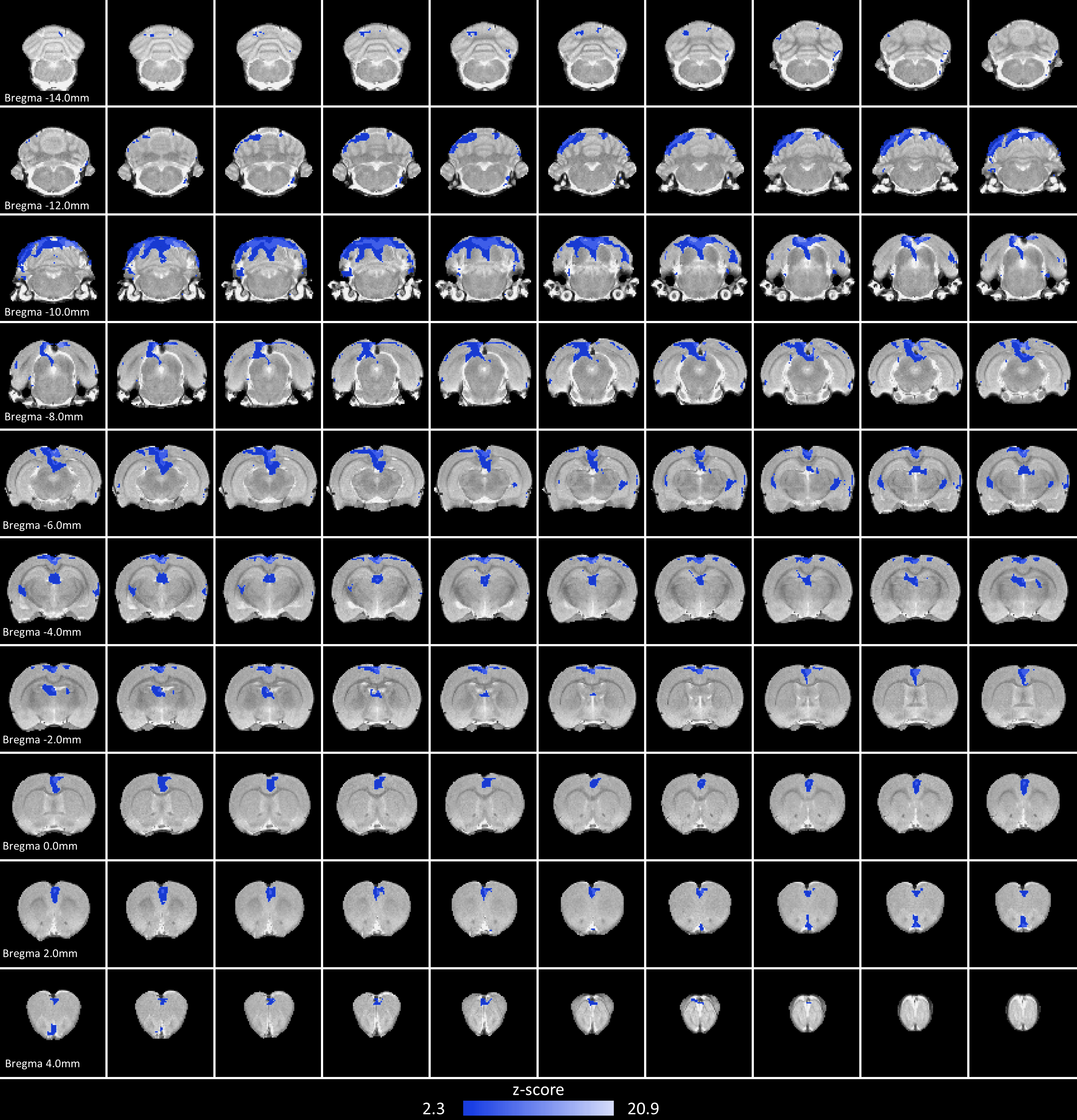
**

**Figure S2:** Whole brain maps showing BOLD signal decreases in animals which received a 2mg/kg dose of psilocin compared to vehicle controls.

| **Stimulation frequency** | **Factor** | **df** | **F** | ***p*** |
| --- | --- | --- | --- | --- |
| 1Hz | Drug treatment | 1,9 | 0.013 | 0.913 |
|  | Pre/post drug administration | 1,9 | 0.041 | 0.843 |
|  | Drug treatment x Pre/post drug administration interaction | 1,9 | 0.004 | 0.952 |
|  |  |  |  |  |
| 2Hz | Drug treatment | 1,9 | 0.256 | 0.625 |
|  | Pre/post drug administration | 1,9 | 0.009 | 0.926 |
|  | Drug treatment x Pre/post drug administration interaction | 1,9 | 0.674 | 0.433 |
|  |  |  |  |  |
| 5Hz | Drug treatment | 1,9 | 1.114 | 0.319 |
|  | Pre/post drug administration | 1,9 | 1.203 | 0.301 |
|  | Drug treatment x Pre/post drug administration interaction | 1,9 | 1.327 | 0.279 |
|  |  |  |  |  |
| 10Hz | Drug treatment | 1,9 | 5.462 | 0.044* |
|  | Pre/post drug administration | 1,9 | 3.359 | 0.100 |
|  | Drug treatment x Pre/post drug administration interaction | 1,9 | 5.125 | 0.050* |
|  |  |  |  |  |
| 20Hz | Drug treatment | 1,9 | 3.187 | 0.108 |
|  | Pre/post drug administration | 1,9 | 0.004 | 0.950 |
|  | Drug treatment x Pre/post drug administration interaction | 1,9 | 3.479 | 0.095 |
|  |  |  |  |  |
| 40Hz | Drug treatment | 1,9 | 2.866 | 0.125 |
|  | Pre/post drug administration | 1,9 | 0.000 | 1.000 |
|  | Drug treatment x Pre/post drug administration interaction | 1,9 | 3.529 | 0.093 |

**Table S1:** Frequency by frequency ANOVA results of comparisons of maximum CBF responses to stimulation. * = *p* < 0.05
